# Quantification and Comprehensive Analysis of Mesenchymal Stromal Cells in Bone Marrow Samples from Sickle Cell Disease Patients with Osteonecrosis

**DOI:** 10.1155/2020/8841191

**Published:** 2020-11-24

**Authors:** Tiago O. Ribeiro, Paula B. Daltro, Gildasio Cerqueira Daltro, Songeli M. Freire, Roberto Meyer, Vitor Fortuna

**Affiliations:** ^1^Health Science Institute, Federal University of Bahia, Salvador, BA 40110-100, Brazil; ^2^Prof. Edgar Santos Hospital Complex, HUPES, Federal University of Bahia, Salvador, BA 40110-060, Brazil

## Abstract

The potential use of bone marrow mesenchymal stromal cells (BM-MSCs) for the treatment of osteonecrosis in sickle cell disease (SCD) patients is increasing. However, convenient BM-MSC quantification and functional property assays are critical factors for cell-based therapies yet to be optimized. This study was designed to quantify the MSC population in bone marrow (BM) samples from SCD patients with osteonecrosis (SCD group) and patients with osteoarticular complications not related to SCD (NS group), using flow cytometry for CD271^+^CD45^-/low^ cell phenotype and CFU-F assay. We also compared expanded BM-MSC osteogenic differentiation, migration, and cytokine secretion potential between these groups. The mean total cell number, CFU-F count, and CD271^+^CD45^-/low^ cells in BM mononuclear concentrate were significantly higher in SCD than in NS patients. A significant correlation between CD271^+^CD45^-/low^ cell number and CFU-F counts was found in SCD (*r* = 0.7483; *p* = 0.0070) and NS (*r* = 0.7167; *p* = 0.0370) BM concentrates. An age-related quantitative reduction of CFU-F counts and CD271^+^CD45^-/low^ cell number was noted. Furthermore, no significant differences in the morphology, replicative capacity, expression of surface markers, multidifferentiation potential, and secretion of cytokines were found in expanded BM-MSCs from SCD and NS groups after *in vitro* culturing. Collectively, this work provides important data for the suitable measurement and expansion of BM-MSC in support to advanced cell-based therapies for SCD patients with osteonecrosis.

## 1. Introduction

Osteonecrosis (ON), a common disabling disorder, affects ∼30% of people with sickle cell disease (SCD), in its early adulthood [1, 2]. The pathogenesis of osteonecrosis presumably involves abnormally adherent sickled erythrocytes to endothelium and repeatedly impaired blood flow to osteochondral bone, causing ischemic death and necrosis of the bone and marrow [3, 4]. Osteonecrosis is initially asymptomatic in SCD patients but may rapidly progress to disabling arthritis due to bone collapse, joint pain, and significant morbidity. Indeed, treatment interventions for early-stage osteonecrosis should delay the progression and preserve the native joint [5]. Accordingly, cell therapy with autologous bone marrow aspirates or concentrates, which contains both hematopoietic and mesenchymal stromal cells (BM-MSCs) in addition to other cell types that may play a role in tissue regeneration, represents a viable alternative for osteonecrosis in SCD [6, 7].

Several studies have reported the biological mechanisms underlying BM-MSC-based therapies in SCD. Lebouvier et al. recently demonstrated that BM-MNCs from SCD patients were viable, highly proliferative, and able to differentiate into functional bone-forming osteoblastic cells in ectopic implantation murine models [8]. Furthermore, the immunoregulatory potential of MSCs from SCD patients was functionally comparable with MSCs from healthy volunteers, produced immunosuppressive factors such as indoleamine 2,3-dioxygenase, and activated immunomodulatory pathways [9], which are important for balanced immune response and successful bone healing. In addition, BM-MSC from SCD patients secreted trophic factors and angiogenic cytokines, causing the formation of new blood vessels [10], which may subsequently improve osteogenesis and tissue regeneration. Thus, these characteristics make BM-MSCs promising candidates for enhancing bone healing and tissue regeneration particularly in complicated conditions such as osteonecrosis in SCD patients.

In most clinical studies for bone regeneration, the efficacy of BM aspirates or concentrates depends on the quantity and quality of implanted BM-MSCs. However, native BM-MSCs are usually applied without the quality assessment before transplantation. The quantitative assessment of bone marrow samples is crucial to compare the clinical outcome between studies and improve the consistency of BM-MSC-based therapies [11–14].

Traditionally, BM-MSCs can be identified by their plastic adherence and ability to form colony-forming unit fibroblasts (CFU-Fs) *in vitro*. The CFU-F assay is commonly performed as an indicator of sample quality and to facilitate the quantitative assessment of BM-MSC. However, it is time-consuming, dependent on the culture conditions, and inappropriate for the routine use before cell culturing. Alternatively, flow cytometry for CD271^+^ cell population has been applied as a rapid and simple detection method of BM-MSC content [15–19].

CD271, known as low affinity nerve growth factor receptor (LNGFR) or p75NTR (neurotrophin receptor), is a cell surface marker that potentially defines a subset of MSC [20, 21]. Many studies have used CD271^+^ expression, in combination with other markers such as CD45, to quantify and sort MSCs from different tissue sources and pathologies [22–24]. CD271 has been proposed to be a versatile marker to identify a BM-MSC population with increased lymphohematopoietic activity and osteo/chondro differentiation potential [20, 25]. Ghazanfari and colleagues recently demonstrated that CD271^+^ MSCs display a different phenotypic, genetic, and epigenetic profile in comparison to cultured MSCs [26]. Furthermore, the combination of CD271^+^CD45^-/low^ allowed to identify a population of cells that was highly enriched for CFU-F [27, 28]. Thus, the CD271 marker, although not specific for BM-MSCs, has been shown to detect all CFU-F in normal human bone marrow [29]. Whether CD271 marker is detected in bone marrow from SCD patients and correlates with CFU-F counts is still poorly understood.

The present study explored the BM aspirates and concentrates from SCD patients with osteonecrosis and non-SCD patients undergoing orthopedic surgery for primary osteoarticular complications. We used CFU-F assay and flow cytometry for CD271^+^CD45^-/low^ phenotype to quantify BM-MSCs and compared their frequency with donor-matched BM aspirates and concentrates. We also used functional in vitro assays for MSC expansion, osteogenic differentiation, migration, and secreted cytokines to demonstrate that MSCs from SCD patients with osteonecrosis were equal or superior to their non-SCD counterparts. These findings should contribute to optimize the autologous BM-MSC-based regenerative therapies for SCD patients.

## 2. Materials and Methods

### 2.1. Patient Selection and Harvest of BM Aspirate

The study was reviewed and approved by the institutional review board of the Health Science Institute (Federal University of Bahia, approval no. 67238317.0.0000.5662). All volunteers gave written informed consent before participation.

Between August 2018 and November 2019, bone marrow (BM) aspirates were obtained from 51 patients (24 male and 27 female) attending the outpatient facility and undergoing elective orthopedic surgery at the Prof. Edgar Santos Hospital Complex (Federal University of Bahia). Age ranged from 18 to 74 years (median 32 years). In this series, bone marrow aspirates were obtained from sickle cell disease patients with osteonecrosis (SCD group, *N* = 32) and from nonsickle cell disease patients (NS group, *N* = 19), undergoing orthopedic surgery for primary osteoarticular complications. The etiology and patients' characteristics are listed in the Online Supplementary Table S1.

Autologous BM aspirate (BMA) was obtained by posterior superior iliac crest aspiration as previously described [30] and used immediately upon receipt. The frequency of nucleated cells in BMA was measured manually by dilution with Turk's solution and counting on a hemocytometer. Inclusion criteria were patients treated in our institution with percutaneous autologous bone marrow transplantation for the treatment of osteoarticular complications. Exclusion criteria were patients with bone inflammation, immunosuppressive drug therapy, metabolic disease, systemic illness, or neoplastic disease.

### 2.2. Bone Marrow Mononuclear Cell (BM-MNC) Concentrate

BM-MNCs were isolated from the BM aspirate (~20 mL) on a Ficoll density gradient (1.077 g/mL) to reduce erythrocyte contamination, according to the instructions of the manufacturer (GE Healthcare, Biolab nordeste, Brazil). Briefly, BM aspirates were diluted 1 : 2 in phosphate buffered saline PBS and centrifuged for 30 minutes on Ficoll separating solution at 400 g. The mononuclear fraction (BM-MNC) was carefully collected and further washed twice in Dulbecco's modified Eagle's medium (DMEM; Sigma-Aldrich, Brazil). The final product consisted of 5 mL BM-MNC suspension; it was stored at room temperature until use. Total leukocytes (WBC) and cell viability were determined by Trypan blue exclusion in a hemocytometer.

### 2.3. Flow Cytometry for BMA and BM-MNCs

To analyze the expression of specific surface proteins, 50 *μ*L BMA or BM-MNCs (2 × 10^5^ cells) resuspended in 0.9% saline solution were placed in FACS tube (Falcon) containing fluorochrome-conjugated monoclonal antibodies and incubated for 30 min at room temperature. The phenotypic identification and frequency assessment of the “ex-vivo MSC” using the CD271^+^CD45^-/low^7AAD^−^ phenotype was performed as previously described (see supplementary figure S1 in the Supplementary Material) [31]. The frequency of viable CD34^+^ cells was evaluated using a gating strategy based on the basic ISHAGE protocol [32]. The appropriate combination of antibodies used at the manufacturers' recommended concentrations was as follows: anti-CD45 antibody (FITC, clone 2D1, mouse IgG1 *κ*, Exbio), anti-CD271 antibody (PE, clone REA648, mouse IgG1, Miltenyi Biotec), anti-CD34 antibody (PE, clone 581, mouse IgG1 *κ*, Exbio), and anti-CD45 antibody (FITC, clone 2D1, mouse IgG1 *κ*, Exbio). 7-Aminoactinomycin-D (7AAD, BD Pharmingen) was added simultaneously in order to distinguish between live and dead cells. After staining, erythrocytes were lysed with 1 mL lysing buffer (Excelllyse Easy; Exbio) at RT for 5 min and analyzed immediately using FACSCalibur. A minimum of 50,000 events was collected for each sample. Unstained and single antibody-stained controls were used to optimize the cytometer voltage settings and spectral compensation, and isotype controls (BD Pharmingen) were used. Absolute cell count was generated by incorporating the leukocyte count from an automated hematology analyzer (two-platform method) as described [31, 33]. Flow cytometry data analysis was performed using the Cell Quest software (BD Biosciences).

### 2.4. CFU Assay

Colony-forming unit fibroblast (CFU-F) assays were performed as described previously [31]. Briefly, 100 *μ*L BMA were seeded into duplicate 35-mm diameter 6-well plates containing 3 mL Dulbecco's modified Eagle's medium (DMEM) (Sigma-Aldrich, Brazil) supplemented with 20% fetal bovine serum (Cultilab, Campinas, Brazil), 100 U/mL penicillin, and 100 *μ*g/mL streptomycin (Sigma-Aldrich, Brazil) and incubated in 5% CO_2_ at 37°C. Medium was renewed every third day. BM-MNCs were seeded at 1 × 10^6^ cells/dish and cultured similarly [30]. After 14 days, adherent cells were washed with PBS, fixed with 4% formaldehyde, and stained with 0.05% crystal violet (Sigma-Aldrich, Brazil). Colonies containing ≥50 fibroblastic cells were manually counted under at 10x magnification microscopy. Colonies were counted in replicates and subsequently compared as mean data for each condition and donor. The concentration of the CFU-F/mL of bone marrow was calculated based on the seeding number and the initial concentration of WBC [34].

### 2.5. MSC Isolation, Culturing, and Expansion

MSCs were cultured as previously described [10]. BM-MNCs were plated in complete Dulbecco's modified Eagle's complete medium (DMEM, low glucose, 10% fetal calf serum and 100 U/mL penicillin/streptomycin) at 100,000 to 300,000 cell/cm^2^. The medium was replaced after four days of culturing, and MSCs were allowed to expand for 7-12 days. MSCs were passaged weekly, and passages 3–6 were used in experimentation. To compare growth expansion between SCD-MSC and NS-MSC, 2.5 × 10^4^ cells were seeded in triplicate into the 6-well plates in complete DMEM (Sigma-Aldrich, Brazil), and viable cell numbers were monitored for 7 days.

### 2.6. Immunophenotyping of Expanded Mesenchymal Stromal Cells and Differentiation Capacity

MSCs (passage 3–6) were stained with monoclonal fluorescein isothiocyanate- and phycoerythrin-conjugated antibodies and analyzed using FACSCalibur (BD Biosciences). The following antibodies were used: CD29-FITC (clone TS2/16), CD90-FITC (clone eBIO5E10), CD105-PE (clone SN6), and anti-CD146-PE (BD Pharmingen®). Fluorochrome-conjugated mouse immunoglobulins were used as isotype controls. For adipogenic and osteogenic multilineage differentiation capacity, MSCs (passages 3-6) were seeded into 6-well plates (osteogenic and adipogenic seeding density of 2 × 10^4^ cell/well) and cultured in adipogenic or osteogenic-inducing medium as previously described [30]. Differentiation medium was changed every 3 days. After 21 days, cell monolayers were fixed in 4% paraformaldehyde (PFA) for 15 min at room temperature. Alizarin red S (Sigma-Aldrich) was used to detect mineralized matrix deposition (an early indicator of osteogenic differentiation), and lipid droplets (indicator of adipogenic differentiation) were detected with Oil Red O solution (Sigma-Aldrich, Brazil). For chondrogenic differentiation, MSCs at passage 3 were pelleted and induction was performed in a modified high-density “micromass” culture using a Chondrogenesis Differentiation Kit (Gibco) in accordance with the manufacturer protocol. Culture medium was changed every 3 days for 21 days. For histological examination, briefly, pellets were fixed overnight in 10% formalin and processed according to the standard procedures for sample processing, embedding, and sectioning. Chondrogenic differentiation was assessed by Alcian blue staining at pH 1.0, 4 weeks after initial chondrogenic induction. The stained sections were mounted with mounting medium (DAKO) and visualized with a light microscope (Eclipse TS100, Nikon).

### 2.7. Indirect Immunofluorescence Assay

SCD-MSC and NS-MSC (2×10^4^ cell/well) grown on glass cover slips were fixed with 4% paraformaldehyde in PBS (pH 7.4) for 15 min at room temperature and permeabilized with 1% Triton X-100 (Sigma-Aldrich, Brazil). Cells were incubated with the following primary antibodies in 3% calf serum, 0.1%Triton X-100 in PBS (pH 7.4): rabbit anti-vimentin (1 : 200; Vector Laboratories) and mouse anti-SMA (1 : 200; Vector Laboratories). Cells were washed and incubated with secondary antibodies goat anti-rabbit Alexa Fluor 555 (Invitrogen) and goat anti-mouse Alexa Fluor 488 (Invitrogen) at 1 : 300 dilution. Nuclei were counterstained with DAPI (4,6-diamidino-2-phenylindole, 1 : 1000). Slides were examined with fluorescence confocal microscope (Leica TCS SP5 software; Leica Microsystems).

### 2.8. Intracellular Osteocalcin Detection

Osteocalcin intracellular staining was performed as previously described [30]. Briefly, MSCs (2×10^5^ cell/well) were grown in an osteoinductive medium for 10 days. Subsequently, cells were detached with 0.125% Trypsin-EDTA, fixed with 4% paraformaldehyde, and permeabilized with saponin buffer for 10 minutes. Next, cells were incubated with mouse anti-human osteocalcin- (OCN-) PE (R&D System) at 4°C for 40 minutes and immediately analyzed using a FACSCalibur flow cytometer (FACSCalibur, BD Biosciences) and a Cell Quest software (Becton Dickinson). Results were expressed as median fluorescence intensities (MFIs), corrected for background fluorescence.

### 2.9. Migration Assay in Boyden Chamber

The vertical migration of SCD-MSC and NS-MSC was analyzed using the transwell migration assay in Boyden chambers. Transwell inserts (8 *μ*m pore size; Millipore) were loaded with 5 × 10^4^ cells into the upper chamber, and 500 *μ*L of DMEM supplemented with 10% FBS was added to the lower chambers. The cells were allowed to migrate at 37°C in a humidified incubator in 5% CO_2_ for 48 h. After incubation, nonmigrating cells were removed from the top chamber using a cotton swab and the cells that migrated to the lower surface were fixed with 4% paraformaldehyde for 15 min and stained with 0.1% crystal violet (Sigma-Aldrich, Brazil). Stained cells from four randomly chosen fields were counted under a light microscope. All experiments were performed at least three times.

### 2.10. Enzyme-Linked Immunosorbent Assay (ELISA)

Interleukin-8 (IL-8), stromal-derived factor-1*α* (SDF-1*α*), and transforming growth factor-*β* (TGF-*β*) immunoreactivity present in the supernatants were measured by specific ELISAs. MSCs were seeded at a density of 1 × 10^5^ cells per well in 24-well plates. After 48 hours at 37°C in a 5% CO_2_ incubator, the supernatants were harvested and centrifuged to remove cell debris. ELISA kit (R&D Systems) was performed according to the manufacturer's instructions.

### 2.11. Statistical Analysis

The normal distribution was determined for the data using the Shapiro-Wilk test and the D'Agostino and Pearson goodness of fit test. Nonparametric Mann–Whitney and Kruskal-Wallis tests were used as the statistical methods to compare two and three groups, respectively. Spearman's correlation test was used to analyze correlation coefficients between clinical assessment results and cell factors. All statistical analyses were performed using the GraphPad Prism software (version 6.0). A value of *p* < 0.05 was considered significant.

## 3. Results

### 3.1. Higher Frequency of Clonogenic Stromal Cells in SCD Samples

**Figure 1:**
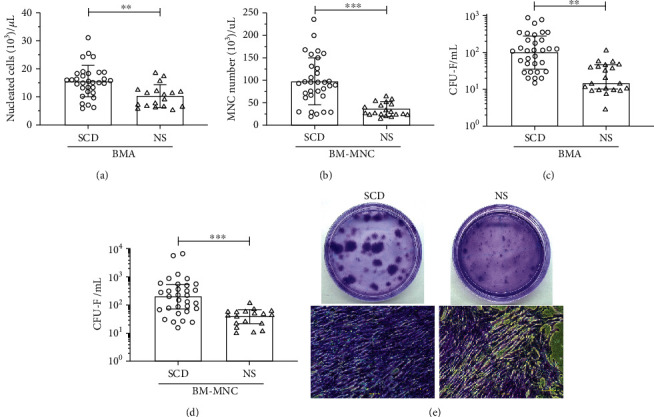
Frequency of CFU-F and BM-nucleated cells. Quantification of (a, b) nucleated cells and (c, d) CFU-F counts in bone marrow aspirate (BMA) or mononuclear fraction (BM-MNC). (e) Typical CFU-F plates from representative donors showing colonies from different sizes after crystal violet staining. Statistical analyses (Mann–Whitney *U* test) were performed between values, and the data are reported as the median and interquartile range. ^∗∗^*p* < 0.01; ^∗∗∗^*p* < 0.005.

Bone marrow aspirates (BMAs) from sickle cell disease (SCD) and nonsickle patients (NS) undergoing orthopedic surgery were collected and immediately processed to isolate mononuclear cells (BM-MNC). The average BMA total cell number and BM-MNC were significantly higher in SCD than in NS group. The BMA on average contained 15.5 ± 5.6 × 10^3^/*μ*L in SCD group and 10.4 ± 4.1 cells × 10^3^/*μ*L in NS group (*p* < 0.01) (Figure 1(a)). After BM processing, a significant larger BM-MNC number was observed in SCD than in NS group (96.7 ± 53.4 vs. 34.8 ± 15.3 cells × 10^3^ cells/*μ*L, *p* < 0.001) (Figure 1(b)). To investigate whether the number of mesenchymal progenitor cells were also increased in BM SCD samples, the clonogenic stromal cells were quantified using the standard CFU-F assay after 14 days in culture. The BMA contained a significant higher number of CFU-F colonies in SCD samples (median 110 CFU-Fs/mL) in comparison to NS samples (15 CFU-Fs/mL) (*p* < 0.01) (Figure 1(b)). After BM processing, the median CFU-F/mL number was 238.2 CFU-Fs/mL (IQR 72–553) in SCD samples compared with 46.5 CFU-Fs/mL (IQR 21–66) in NS samples (*p* < 0.05) (Figure 1(d)). A significant CFU-F enrichment was observed for both SCD (1.96-folds; *p* = 0.02) and NS (3.0-folds; *p* = 0.03) samples after BM processing (Table 1). Consistent with previously reported findings, a high donor-to-donor variation was observed in both groups, potentially due to factors related to donor age or the harvesting technique during the aspiration procedure [19, 34]. Microscopical analysis demonstrated that CFU-Fs exhibited intra- and interdonor heterogeneity in both SCD and NS groups, with either large or small colonies formed by fibroblastoid cells (Figure 1(e)). These findings indicate an effective enrichment of CFU-F in BM-MNC samples from patients with osteonecrosis, which is consistent with previous reports [8, 35].

### 3.2. Frequency of CD271^+^CD45^-/low^ and CD45^dim^/CD34^+^ Cells in SCD Samples

**Figure 2:**
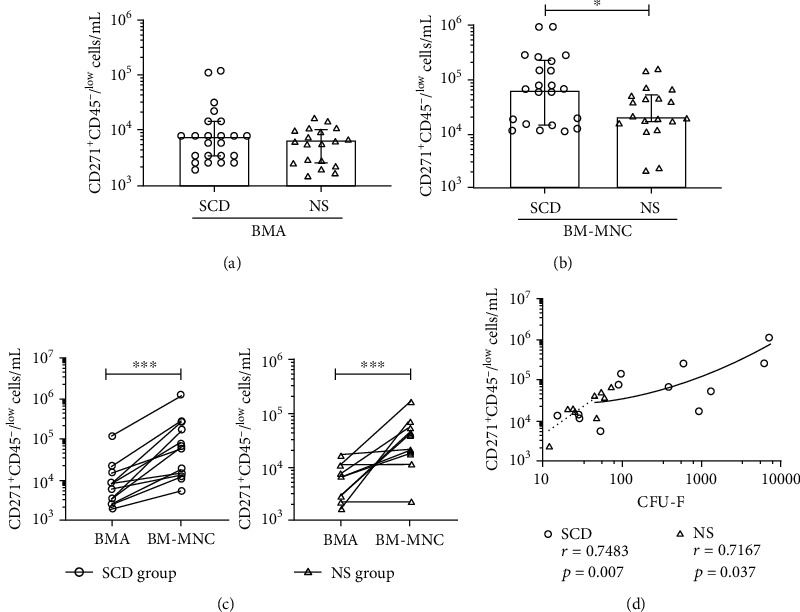
Quantification of CD271^+^CD45^-/low^ cells in BMA and BM-MNC. Counting of CD271^+^CD45^-/low^ cells in (a) BMA and (b) BM-MNC from SCD and NS patients. Data are reported as the median and interquartile range. (c) The fold increase of CD271^+^CD45^low^ cell counts after sample processing in SCD and NS. Wilcoxon matched-pairs signed-rank test. (d) Correlation between the frequency of CFU-F and CD271^+^CD45^-/low^ cell counts. A Spearman *r* test was used for the correlation analysis; black and dashed lines across data sets indicate of best fit for each group. ^∗^*p* < 0.05; ^∗∗^*p* < 0.01; ^∗∗∗^*p* < 0.001.

CFU-Fs in human BMA samples are described as a rare cell population characterized by CD271^+^CD45^-/low^ phenotype [29]. To investigate the frequency CD271^+^CD45^-/low^ phenotype, BM samples were enumerated using flow cytometry. Cell viability was monitored with 7AAD nuclear dye, and a minimal 95% value was obtained. In fresh BMA, the median CD271^+^CD45^-/low^ counts in the SCD group were comparable to that found in the NS group (Figures 2(a) and 2(b) and Table 1). After BM processing, the median CD271^+^CD45^-/low^ cell count was 67.1 × 10^3^ cell/mL (IQR 15.0 to 237.5 × 10^3^ cell/mL) in SCD group while 21.6 × 10^3^ cell/mL (IQR 18.2 to 54.2 × 10^3^ cell/mL) in the NS group, a pattern similar to CFU-F counts, reaching statistical significance (*p* = 0.04) (Figure 2(b)). A significant enrichment in the CD271^+^CD45^-/low^ cell counts was observed for both SCD (mean, 12.6-folds; 95% CI, 1.8 to 44.1-folds; *p* < 0.0005) and NS (mean, 10.3-folds; 95% CI, 1.0 to 43.1-folds; *p* < 0.005) groups after BM processing (Figure 2(c)).

Further, we asked whether the higher frequency of CFU-Fs/mL correlates with the numbers of CD271^+^CD45^-/low^ cell phenotype in each study group. Linear regression analysis revealed a moderate but significant correlation between CD271^+^CD45^-/low^ cell number and CFU-F counts in SCD (*r* = 0.7483; *p* = 0.0070) and NS samples (*r* = 0.7167; *p* = 0.037) after BM processing. These data suggest a possibility of using flow cytometry for quantification of CD271^+^CD45^-/low^ in BM aspirates and enriched BM-MNC fractions of sickle cell disease patients with osteonecrosis.

**Figure 3:**
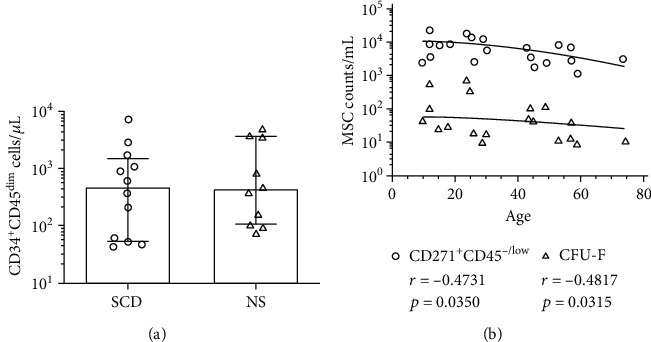
Quantification of hematopoietic progenitors in BM-MNC and correlation of MSC with donor age. (a) Counting of CD45^dim^CD34^+^ cells in BM-MNC. (b) Correlation between putative MSC (either CFU-F frequency or CD271^+^CD45^-/low^ cell counts) and increasing donor age. A Spearman *r* test was used for the correlation analysis independent of osteoarticular complication. Black and dashed lines across data sets indicate of best fit for each group. Statistical analyses (Mann–Whitney *U* test) were performed between values, and the data are reported as the median and interquartile range.

As hematopoietic stem/progenitor cells (HSPC), identified as CD45^dim^/CD34^+^, and MSCs are simultaneously aspirated during bone marrow harvesting [34], we investigated if CD271^+^CD45^-/low^ phenotype could be correlated with HSPC after BM processing. The HSPC counts showed no significant difference between SCD and NS group (Figure 3(a)). After BM processing, the median HSPC concentration was 485.3 cells/*μ*L in the SCD group (IQR 53 to 1479 cells/*μ*L) and 435.1 cells/*μ*L in the NS group (range from 105 to 3763 cells/*μ*L) (Figure 3(a) and Table 1). We did not observe a significant correlation between CD45^dim^/CD34^+^ cells and CD271^+^CD45^-/low^ cell counts in SCD group and NS group (data no shown).

Next, the data from both SCD and NS groups were further analyzed according to the age of the bone marrow donors. Independent of osteoarticular complication, there was a moderate but significant gradual decline in the number of CFU-F colonies (*r* = −0.4817; *p* = 0.0315; *N* = 20) with increasing age of the donor (see supplementary Table S2). This pattern was also consistently observed between the age of the donor and the number of CD271^+^CD45^-/low^ measured by flow cytometry (*r* = −0.4731; *p* = 0.0350; *N* = 20) (Figure 3(b)). In sum, putative MSCs, identified as CD271^+^CD45^-/low^ cell counts, were quickly quantified in BM aspirates and enriched BM-MNC fractions of sickle cell disease patients with osteonecrosis and positively correlated with CFU-F colonies.

### 3.3. Phenotypic Characterization and *In Vitro* Growth Capacity of BM-MSCs

**Figure 4:**
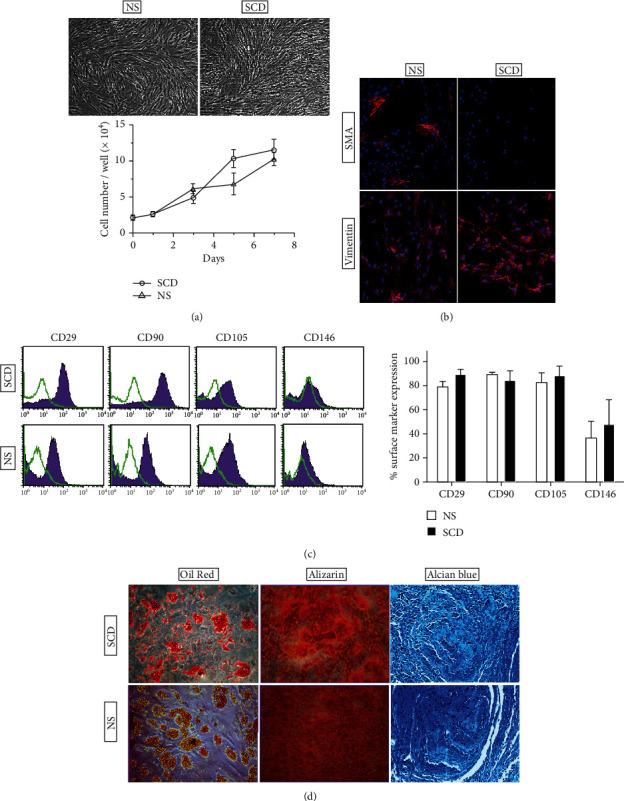
BM-MSCs from SCD and NS patients display similar immunophenotypes and *in vitro* characteristics. (a) Morphology and cell growth expansion of BM-MSC. Cells were subcultured and counted on the indicated day for 7 days. (b) Immunocytochemistry detection shows *α*-SMA and vimentin-positive BM-MSCs. Nuclei were stained with Hoechst dye (blue). (c) Flow cytometry histograms for positive MSC specific markers (purple line) and the respective isotype-matched control (green line) are shown. (d) Multipotential BM-MSCs from representative SCD and NS patients were differentiated toward adipogenic, osteoblastic, and chondrogenic lineages. Accumulation of intracellular lipid vacuoles shown by Oil Red O staining (left), calcium-rich extracellular matrix as evidenced by Alizarin red S (middle), and static micromass cell culture stained with Alcian blue (right). Statistical analyses (Student *t*-test) were performed between values, and the data are reported as the mean and standard deviation. Scale bar = 50 *μ*m.

Our assessment of MSC frequency indicated that the number of CFU-F/mL was increased in BM samples from SCD patients in comparison to NS patients, which is consistent with previous reports [8, 25]. Then, an extended analysis of cultured MSC was undertaken to identify any potential difference in terms of morphology, immunophenotype, multilineage potential, and functional characteristics after *in vitro* culturing and expansion. For the following experiments, only BM-MSC between passages 3 and 6 from young to adult (19-40 years old) donors were included, since previous reports that increasing donor age accelerated changes in BM-MSC morphology and proliferation [19, 36]. BM-MSC from SCD (SCD-MSC) and NS (NS-MSC) patients exhibited similar spindle-shaped fibroblastoid morphology up to the first six passages. In the initial phases of cultivation, we found that the growth ability of SCD-MSC was slightly higher but not significantly different than NS-MSCs (Figure 4(a)). BM-MSC from SCD and NS patients displayed typical mesenchymal markers equally positive for CD29, CD90, and CD105 (Figure 4(c)) while lacking expression of hematopoietic markers (HLA-DR, CD14, CD34, and CD45) (data not shown) over the first six passages, in accordance with the minimal criteria for BM-MSC [37]. Representative markers, such as vimentin and SMA were also detected in both BM-MSCs. No significant differences in the expression of any of these markers were observed between SCD-MSC and NS-MSC (Figure 4(b)). In addition, cultured cells from both groups exhibited similar differentiation potential *in vitro* toward the osteoblastic (Alizarin red staining), chondroblastic (Alcian blue), and adipogenic (Oil Red O staining) lineages (Figure 4(d)).

### 3.4. BM-MSCs from SCD and NS Patients Had Similar Characteristics when Culture Expanded

**Figure 5:**
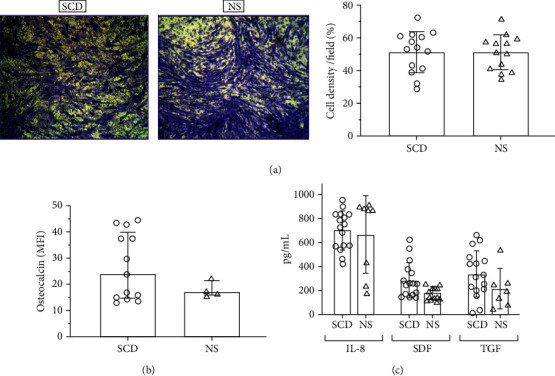
BM-MSCs from SCD and NS patients showed no significant differences in migration, osteogenic marker, and secreted cytokines. (a) Boyden chamber analysis. Uncoated Boyden chambers were used to analyze migration of BM-MSCs. Cells that migrated through the chambers were stained with crystal violet and quantitated (right). (b) Quantification of intracellular osteocalcin expression in BM-MSCs grown under osteoinductive conditions for 10 days. (c) Enzyme-linked immunosorbent assay for interleukin-8 (IL-8), stromal cell-derived factor-1 alpha (SDF-1*α*), and transforming growth factor-beta (TGF-*β*). Concentration (pg/mL) of cytokines and growth factors in supernatants of BM-MSCs. Statistical analyses (Mann–Whitney *U* test) were performed between values, and the data are reported as the median and interquartile range.

Previous studies have indicated that functional characteristics of MSCs are not impaired in patients with osteonecrosis [8, 38], while others have observed a decline in the proliferation rate and osteogenic activity [39–41]. As stem cell-guided migration is a vital step in the bone healing process, we used Boyden chamber assays to compare SCD-MSC and NS-MSC migration in response to serum-mediated chemoattraction. After a 20-hour incubation, migrated cells on the underside of the filter were 10% formalin fixed and quantified with 1% crystal violet staining. As shown in Figure 5(a), both SCD- and NS-MSC exhibit equally a high level of migratory response toward 10% FBS. The negative control samples showed negligible cell migration. Additionally, MSC response to serum was observed in a dose-dependent manner with 15% FBS having the greatest effect (not shown). Next, we investigated the expression level of osteocalcin, a marker of developing osteoblasts. After 10 days in the presence of osteogenic inducers, no significant difference between SDC- and NS-MSC was observed (Figure 5(b)).

MSCs secrete trophic factors and cytokines that reportedly promote cell survival and bone regeneration. Then, we investigated if the levels of IL-8, TGF-*β*, and SDF-1*α* in the supernatant of SCD- and NS-MSCs were also equivalent. We found that BM-MSCs grown under control conditions secreted significant amounts of IL-8, TGF-*β*, and SDF-1*α*, although no significant different levels between SCD and NS-MSCs were observed (Figure 5(c)). These results indicate that BM-MSCs from SCD and NS patients are equally capable of producing tissue repair cytokines and growth factors.

In sum, culturing did not result in any detectable changes with respect to *in vitro* characteristics evaluated in both BM-MSCs from SCD and NS patients.

## 4. Discussion

Cell-based therapies with autologous BM aspirates or concentrates as a source of osteoprogenitors have emerged lately with great popularity for the treatment of osteonecrosis and other osteoarticular injuries in SCD patients. However, the quality of these BM-MSCs is poorly understood, and the pathophysiology associated with SCD may result in their functional impairment and limited repair capacity. In this study, we comprehensively measured the abundance of CD271^+^CD45^-/low^ cell phenotype and compared with CFU-F frequency, the gold standard assay indicative of osteoprogenitors in BM samples. We demonstrated that the prevalence of CFU-Fs was positively correlated to CD271^+^CD45^-/low^ counts in BM preparations from SCD patients with osteonecrosis. Consistent with published studies [8, 9], our data also suggested that BM-MSCs isolated and expanded from SCD patients with osteonecrosis were equivalent to BM-MSCs from the control groups in terms of their phenotypic and functional properties. Collectively, this work provides important data for the quick measurement of putative BM-MSC in support to advanced cell-based therapies for SCD patients with osteonecrosis.

To our knowledge, this study is the first to evaluate CD271^+^CD45^-/low^ cell phenotype in SCD patients with osteonecrosis. CD271 has been proposed as one of the characteristic markers of native BM-MSCs, with prominent osteogenic activity [21, 42]. Their gene expression profile indicated a predilection for bone formation as evident by the elevated levels of numerous osteogenic-lineage molecules [25]. Several independent studies have confirmed that CD271^+^CD45^-/low^ cells are often abundant in fresh BM samples from adult healthy donors [16, 31, 43, 44] but its presence has been less extensively researched in systemic or hematologic diseases. Here, we showed that the number of CD271^+^CD45^-/low^ cells was highly variable but comparable between SCD and control samples. The variability observed herein could not be correlated with sex or disease condition but was consistent with the findings of other works [19]. Alvarez-Viejo et al. reported a low percentage (0.0042%) and high variability of CD271^+^CD45^−^ cell phenotype in the BM aspirates of diabetic patients with foot ulcers compared to healthy donors [11]. Conversely, in chronic osteoarthritis patients, the frequency of CD271^+^CD45^low^ cells, their telomere status, and osteogenic abilities were similar to that in healthy individuals [45]. Among healthy patients who underwent orthopedic surgery, the prevalence of CD271^+^CD45^−^ cells was also described as highly variable [34]. Such variability could be explained by different BM harvesting protocols, different enumeration methods, or limited number of samples [17, 34].

To date, there is no standardized enumeration method for BM-MSCs before cell culturing. Instead, a cytometric assay for CD271^+^CD45^-/low^ phenotype has been used reliably to predict the frequency of BM-MSC in BM samples [11, 16]. However, this correlation has not been demonstrated in BM preparation from SCD patients with osteonecrosis. In our study, we observed a positive correlation between the CD271^+^CD45^-/low^ cell phenotype and CFU-F counts in SCD and NS groups after BM concentration. Our results demonstrated that after bone marrow processing, the CD271^+^CD45^-/low^ cell counts and CFU-Fs could be significantly enriched. Compared with our data, El-Jawhari et al. showed a strong linear correlation between the enrichment of CD271^+^CD45^low^ cell counts and CFU-Fs in bone marrow concentrates [16]. Furthermore, findings by Rebolj and colleagues suggested that the CD271^+^CD45^−^ cell population correlates better with CFU-F numbers than another more stringent MSC phenotype, such as CD45^−^CD73^+^CD90^+^CD105^+^ cells [34]. Another recent study showed that commercial BM processing systems produced CFU-F and CD271^+^CD45^low^ enrichment between 4.4- and 41.2-folds, but dissimilar levels of growth factors and hematopoietic progenitors [46]. These results emphasize the potential value of CD271^+^CD45^-/low^ measurement to predict the frequency of MSC content in BM concentrates for cell-based therapies. The cytometric assessment of CD271^+^CD45^-/low^ cells on routine practice would ensure that a therapeutic dose of MSCs could be adjusted before implantation, which is currently not possible.

The mean concentration of CD271^+^CD45^-/low^ cells/mL measured with flow cytometry in our study was much higher than the concentration of MSC predicted with CFU-F assay. We noted 70 times more CD271^+^CD45^-/low^ cells/mL with the flow cytometric method than putative MSCs quantified by CFU-F assay. This suggests that not all CD271^+^CD45^-/low^ cells could form colonies, which is in agreement with previous studies [19, 47]. Also, we have followed optimal and consistent culture conditions by using complete media and batch-tested bovine serum for the CFU-F assays. Thus, the possibility of the underestimation of CFU-Fs is small, but still exists.

Previous independent studies have documented an age-related decline in BM-MSC numbers, measured either as CFU-F or CD271^+^CD45^−^ counts, in healthy individuals [16, 19, 34]. Based on these results, some studies have suggested that the amount of harvested bone marrow should be adjusted according to the age of the patient, in order to achieve a target BM-MSC number for bone repair [34, 48]. In our study, we have observed a slightly inverse correlation between donor age and the BM-MSC numbers in fresh samples. Given the small number of patient samples evaluated in our study and the large donor-to-donor variability, additional studies are needed to validate our results.

We next focused on the *in vitro* culturing and expansion in order to identify any potential difference in terms of immunophenotypic and functional characteristics of BM-MSCs. No significant differences in the morphology, expansion capacity, expression of surface markers, multidifferentiation potential, and secretion of cytokines were found in BM-MSCs from SCD and control group samples, which is consistent with previous reports [8, 38, 49]. In contrast, a decreased osteogenic ability and enhanced adipogenesis were demonstrated in native BM-MSC from patients with corticosteroid-related osteonecrosis [40, 41]. In our case series, this comparison was not made because none of the patients suffered from corticosteroid-induced osteonecrosis.

Accumulated evidence has indicated that the therapeutic benefit of MSCs is attributable not only to their differentiation potential but also to their secreted factors [50, 51]. In line with this, many reports have demonstrated the transplantation of MSC-derived secretome-enhanced blood vessel regeneration and bone reconstruction in a preclinical model of osteonecrosis and bone defects [52–54]. Our study demonstrated that SCD-MSC and NS-MSC under standard culture conditions secreted high levels of IL-8, TGF-*β*, and SDF-1*α*; cytokines involved with bone tissue [55–57].

The primary limitation of this study is related to sample size and age distribution. BM samples from NS patients were not routinely obtained, which resulted in a smaller sample size and older patients in this group.

In conclusion, the results presented here showed that the quantification of CD271^+^CD45^-/low^ cell phenotype was a fast and suitable approach to predict MSC number, with positive correlation with CFU-Fs in SCD BM concentrates. In relation to possible therapeutic applications of cultured BM-MSCs, our data indicated that expanded BM-MSC did not have their functional properties impaired in regard to multipotential, proliferative, migration, and paracrine ability. Thus, this work provides important preclinical data that is necessary to help indicate the “number” of MSCs in bone marrow samples prior to their use in cell-based therapies for SCD patients with osteonecrosis.

## Figures and Tables

**Table 1:** Distribution of cell number, progenitor cell populations, and frequency of CFU-F with BM processing.

	BMA			BM-MNC		
	SCD	NS	*p*	SCD	NS	*p*
Cell number × 10^3^/*μ*L (mean ± SD)^§^	15.5 ± 5.6	10.4 ± 4.1	<0.01	96.7 ± 53.4	34.8 ± 15.3	<0.001
CFU-F/mL	110 (34-278)	15 (10-45)	<0.01	216 (72-553)	46 (21-66)	<0.001
CD271^+^CD45^−/low^ × 10^3^/mL	7.8 (3.4–14.4)	6.7 (2.9–11.0)	>0.05	66.7 (14.2–260)	21.6 × (18.2‐54.2)	<0.05
CD34^+^CD45^low^ × 10^3^/*μ*L	155 (120.5-189.5)	126 (94-173)	>0.05	485.3 (53.4 -1479)	435.1 (105-3763)	>0.05

Demographic and baseline biochemical characteristics of patients. Variables presented as median (interquartile range). ^§^Except where noted otherwise. Abbreviations: BMA: bone marrow aspirate; BM-MNCs: bone marrow mononuclear cells; CFU-Fs: colony-forming unit fibroblasts.

## Data Availability

The data used to support the findings of this study are available from the corresponding author upon request. Data are available from the internal database of Health Science Institute, Federal University of Bahia, Bahia, Brazil, upon Ethics Committee approval for researchers who meet the criteria for access to confidential data.

## Supplementary Materials

Supplementary materials Supplementary Table S1: clinical and biochemical characteristics of patients. Supplementary Table S2: data with MSC count/mL respective to Figure 3(b). Supplementary Figure S1: gating strategy for the frequency of CD271^+^CD45^-/low^ cell phenotype.
