# Supplementary material for: Quantification and Comprehensive Analysis of Mesenchymal Stromal Cells in Bone Marrow Samples from Sickle Cell Disease Patients with Osteonecrosis
Source: Stem Cells Int. 2020 Nov 24;2020:8841191. doi: 10.1155/2020/8841191 (PMC7710439; doi:10.1155/2020/8841191)
Supplement: Supplementary materials — Supplementary Table S1: clinical and biochemical characteristics of patients. Supplementary Table S2: data with MSC count/mL respective to Figure 3(b). Supplementary Figure S1: gating strategy for the frequency of CD271+CD45-/low cell phenotype. [file 8841191.f1.pdf]

Supplementary Table S1. Clinical and biochemical characteristics of patients

Parameter	SCD	NS	P value
**Nº of individuals**	32	19	
**Average Age of the donors**	28.4 (10 – 55)	46.9 (24 – 74)	
**Male**	13 (40.62 %)	11 (57.90 %)	
**Female**	19 (59.38 %)	8 (42,10 %)	
**Localization**			
**Femoral Head**	16	9	
**Knee**	6	0	
**Hip**	16	4	
**Hematological Data**			
**Hb (g/L)**	10.08 ± 1.78	11.90 ± 3.81	0.0054
**Hematocrit (%)**	17.90 ± 11.99	37.11 ± 8.09	< 0.0001
**Platelel count (x 10^3/ µL)**	342.4 ± 154.9	168.6 ± 79.82	< 0.0001

Demographic and baseline biochemical characteristics of subjects are presented in Supplementary Table. All variables presented as mean (range), n (%) or mean (standard deviation). Abbreviations: Hb, Hemoglobin.

Supplementary Table 2. Data with MSC count/mL respective to Fig 3b

Patient	Age	Gender	Group	$CD271^{+}CD45^{-/low}$	CFU-F
**1**	10	F	SCD	2480	50
**2**	15	F	SCD	7800	30
**3**	12	M	SCD	7880	595
**4**	12	F	SCD	21870	110
**5**	12	F	SCD	3480	110
**6**	18	F	SCD	8140	30
**7**	24	F	NS	16800	760
**8**	25	M	SCD	14410	360
**9**	26	M	SCD	2550	20
**10**	29	M	NS	11280	10
**11**	30	F	SCD	5820	20
**12**	43	M	NS	6660	50
**13**	44	F	SCD	3456	120
**14**	45	M	NS	1710	40
**15**	49	F	NS	2320	120
**16**	53	F	NS	7840	12
**17**	55	F	SCD	6580	40
**18**	57	F	NS	2960	15
**19**	59	M	NS	1106	10
**20**	74	F	NS	3050	12

Abbreviations: F, female; M, male, CFU-F, Colony-forming unit fibroblast; MSC, mesenchymal stromal cells

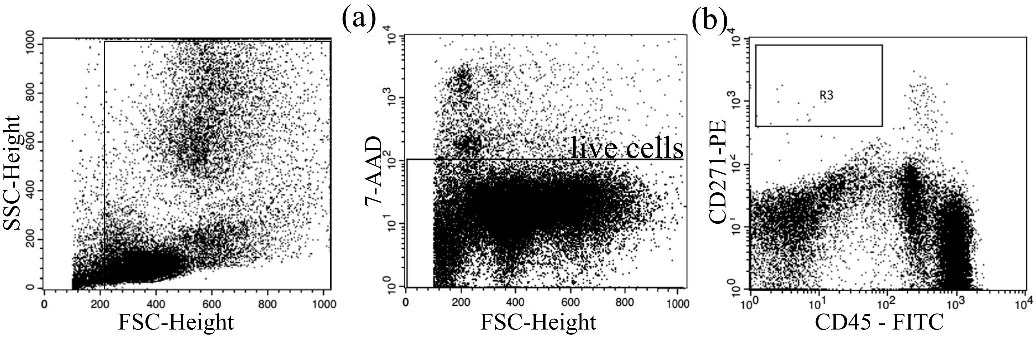

Supplementary Figure S1: Gating strategy for the frequency of $CD271^{+}CD45^{-/low}$ cell phenotype. Cell debris are removed from the analysis based on forward scatter. (a) Dead cells are gated out based on the uptake of 7-AAD. (b) Frequency assessment of $CD271^{+}CD45^{-/low}$ cells are based on a simple rectangular gate.
